# Increase in Lead (Pb) Concentration in the Soil Can Cause Morphophysiological Changes in the Leaves of *Inga vera* subsp. *affinis* (DC.) T.D.Penn. and *Inga laurina* (Sw.) Willd.

**DOI:** 10.3390/plants14060856

**Published:** 2025-03-10

**Authors:** Isabella Fiorini de Carvalho, Patricia Fernanda Rosalem, Caroline de Lima Frachia, Patrícia Borges Alves, Bruno Bonadio Cozin, Ricardo de Almeida Gonçalves, Nayane Cristina Pires Bomfim, Roberta Possas de Souza, Aline Redondo Martins, Liliane Santos de Camargos

**Affiliations:** Physiology of Plant Metabolism Laboratory and Studies in Plant Morphology and Anatomy Laboratory, Department of Biology and Zootechny, School of Engineering, São Paulo State University (UNESP), Ilha Solteira 15385-007, Brazil; if.carvalho@unesp.br (I.F.d.C.); patricia.rosalem@unesp.br (P.F.R.); ca_frachia@yahoo.com.br (C.d.L.F.); patricia.borges@unesp.br (P.B.A.); bruno.bonadio-cozin@unesp.br (B.B.C.); ricardo.goncalves@unesp.br (R.d.A.G.); nayanecristinapires@gmail.com (N.C.P.B.); roberta.possas@unesp.br (R.P.d.S.); aline.martins@unesp.br (A.R.M.)

**Keywords:** abiotic stress, gas exchange, heavy metal, leaf anatomy, legume, morphophysiology, photosynthesis

## Abstract

The accumulation of heavy metals, such as lead (Pb), causes environmental degradation, affecting human health and plant metabolism. Pb can alter plant physiological processes, including photosynthesis, influencing the structure of chloroplasts and leaf tissues. The present study aimed to evaluate the effect of increasing lead concentrations in soil on gas exchange, photosynthetic pigments, and the anatomy of leaf tissues in *Inga vera* subsp. *affinis* and *Inga laurina*. The experiment was conducted in a greenhouse using a randomized block design in a 2 × 6 factorial scheme, with Pb concentrations of 0, 100, 200, 300, 400, and 500 mg dm^−3^. *I. vera* subsp. *affinis* and *I. laurina* maintained stable photosynthetic parameters even under high Pb concentrations. Regarding photosynthetic pigments, *I. vera* subsp. *affinis* exhibited high levels of chlorophyll a and b, even at the highest Pb concentration. Additionally, *I. laurina* showed a greater accumulation of carotenoids and phenolic compounds at higher Pb doses. In leaf tissues, Pb did not alter thickness. These results suggest that both species possess adaptation mechanisms to heavy metal stress, enabling the maintenance of photosynthetic activity and ensuring the completion of their life cycle under adverse conditions.

## 1. Introduction

One of the main problems related to the accumulation of heavy metals is the fact that they interact with a vast number of molecules that are not metabolizable, that is, that accumulate in living organisms [1]. Furthermore, heavy metal accumulation causes extensive degradation of the contaminated area, affecting flora, fauna, and surface and groundwater, altering natural cycles and biochemical processes, causing problems for human health [2].

Among the metals commonly found in cases of soil contamination is lead (Pb), one of the oldest toxic elements and considered the metal with the greatest human interaction [3]. As it is a non-essential element for plants, excess Pb can lead to several negative effects, such as reduced growth, chlorosis, inhibition of photosynthesis, changes in mineral nutrition, water status, and hormonal balance [4]. Furthermore, lead can affect membranes’ structure and permeability, resulting in morphological, physiological, and biochemical impacts on plants [4].

Soil pollution by lead is one of the most worrying environmental challenges, being widely examined in several studies on its contamination levels [5]. A balanced soil provides an environment suitable for the survival of organisms and the functioning of biological and chemical processes. However, the presence of this pollutant can compromise this dynamic, resulting in the manipulation of soil quality. Furthermore, plants exposed to high concentrations of lead may suffer damage to growth and development [6]. Studies on the assimilation of heavy metals by plants show that this metal can have adverse effects on plant growth and seed germination, in addition to restricting chlorophyll production, causing damage to genetic material and causing changes in enzymatic functioning [7].

The presence of heavy metals, especially lead (Pb), has a significant impact on plant gas exchange, negatively influencing growth and development. After absorption, Pb can be translocated to different plant organs, interfering with biochemical and molecular processes essential for plant physiology [8].

Exposure to Pb can result in a reduction in photosynthetic rate and stomatal conductance, impairing the efficiency of the photosynthetic apparatus. Additionally, the stress induced by this metal promotes the production of reactive oxygen species (ROS), which can cause oxidative damage to cell membranes and organelles, including chloroplasts. These effects compromise the plant’s photosynthetic capacity, reducing biomass production and consequently hindering its growth and development [8].

The leaf is an extremely important organ, as it is where photosynthesis occurs, which ensures the functioning of all plant tissues; therefore, structural modifications can affect this vital process [9]. In the case of Pb, an increase in the amount of starch granules was reported in plants subjected to doses of 10^−5^, 10^−4^, and 10^−3^ M of Pb (NO_3_)_2_, and this increase was considered an indication of photosynthetic efficiency [10]. However, the ultrastructural response of each species may vary, and Pb may cause impacts, such as a decrease in the number of thylakoids per granum, at doses between 0.5 and 0.8 mM Pb (NO_3_)_2_ [11], and degradation of cells containing chloroplasts, reducing chloroplast size and compressing thylakoids in the grana, in treatments with 80 and 160 mg of Pb (NO_3_)_2_ [12].

In leaf tissues, studies show that Pb (100 µM Pb^2+^) can reduce the central vein length and width, cause disorganization in the xylem and phloem [13], and decrease the diameter of the vessel elements, starting from 200 mg kg^−1^ soil with Pb(NO_3_)_2_ in combination with another metal such as Ni [14]. On the leaf surface, Pb caused significant variations related to stomata, such as an increase in number, stomatal density, and decrease in width, and induced the closure of most stomata on the abaxial surface [13].

In another case, this metal has increased the secretory ducts’ diameter at low doses (0, 0.25 mM Pb(NO_3_)_2_) and decreased it at high doses (0.5, 1.0 mM Pb(NO_3_)_2_), having even influenced the increase in essential oil production [15], demonstrating a positive response for oil production; in addition, it causes different responses for each plant species. Therefore, anatomical studies analyzing mesophyll tissues, such as palisade and lacunar parenchyma that concentrate chloroplasts, are essential to understand whether metals can affect the photosynthetic process [9].

An interesting family to conduct studies of this type may be Fabaceae, which represents the third largest family of flowering plants, encompassing about 765 genera and 19,500 species [16]. These plants are known for their ability to fix atmospheric nitrogen in a symbiotic relationship with rhizobia bacteria, which contributes to improving soil fertility and reducing dependence on synthetic fertilizers [16].

*Inga laurina* (Sw.) Willd. and *Inga vera* subsp. *affinis* (DC.) TDPenn. are two species of legumes belonging to the genus *Inga*, a genus that contains around 300 species in neotropical regions; around 140 occur in Brazil [17]. *Inga laurina* is a tree species with cylindrical to angular branches, compound petiolate leaves, and spike inflorescences with sessile flowers, whose flowering period occurs between September and November, and fruiting from December to January [18]. While *Inga vera* subsp. *affinis* is a tree species with a straight or slightly grooved trunk, paripinnate leaves with three to six pairs of leaflets, inflorescences in spikes with white, hairy flowers, with a variable flowering period (usually between August and November, and December and April), and fruiting in September and between December and February [19]. The species *Inga vera* subsp. *affinis* and *Inga laurina* have ecological and physiological characteristics that are suitable for studies on responses to heavy metal stress. Both occur naturally in riverine areas and secondary forests, demonstrating tolerance to degraded soils and variations in fertility. As legumes, they have symbiotic associations with nitrogen-fixing bacteria, which improve soil fertility and can increase soil resilience to chemical stress. Furthermore, studies revealed that *Inga vera* subsp. *affinis* presents initial tolerance and phytoremediation potential in soils contaminated with lead, while *Inga laurina* showed relatively low values of bioaccumulation of this metal and low translocation to the aerial part, with higher concentration in the roots, revealing a tolerance strategy based on the confinement of the metal in the underground parts. This behavior can minimize toxic effects on the most sensitive parts of the plant and reduce the dispersion of contaminants in the food chain. Furthermore, its resistance to different environmental conditions, such as variations in humidity and shading, and its relatively fast growth with high biomass production, favor its use in recovery programs for degraded areas. Thus, these species can be used both as indicators of environmental contamination and in phytoremediation strategies, helping to recover soils affected by heavy metals, such as lead [20,21]. However, there is no data on possible morphophysiological changes in response to lead for these species.

Thus, this study aimed to evaluate possible morphophysiological changes in the leaf tissues of *Inga vera* subsp. *affinis* and *Inga laurina* under high concentrations of Pb in the soil, such as anatomical changes in the leaves, gas exchange, and photosynthetic pigments.

## 2. Results

### 2.1. Photosynthetic Parameters

#### 2.1.1. Transpiration (E)

**Figure 1:**
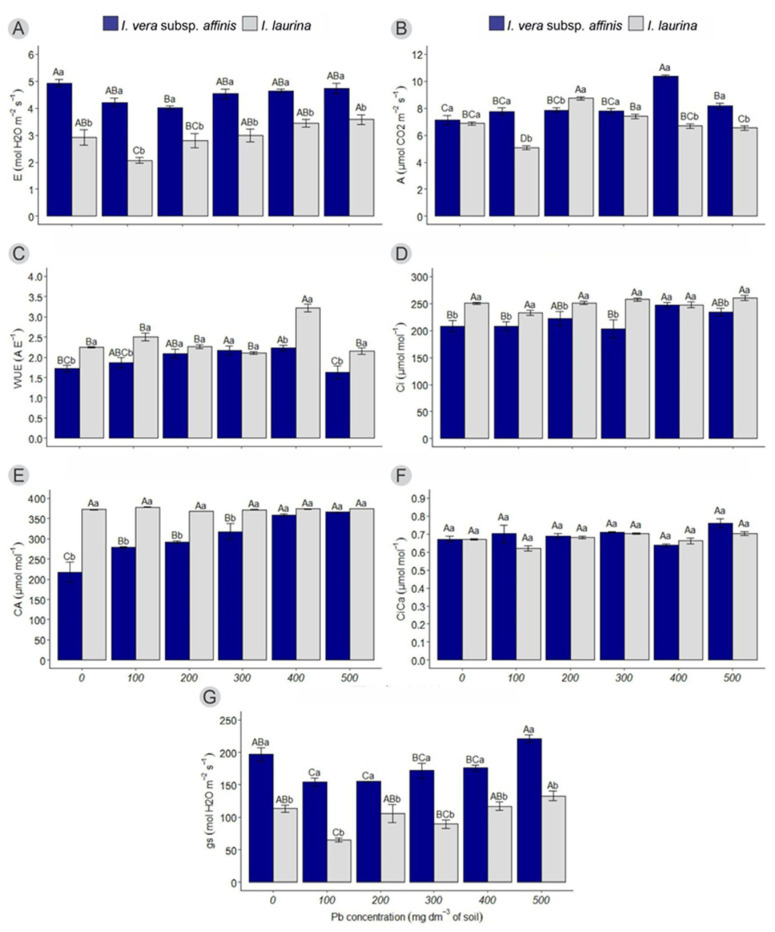
The average of treatments at different lead concentrations for (**A**) transpiration, (**B**) photosynthetic rate, (**C**) water use efficiency, (**D**) internal CO_2_ concentration, (**E**) external CO_2_ concentration, (**F**) ratio between external CO_2_ concentration, and (**G**) stomatal conductance. Different letters indicate a significant difference by Tukey’s test (*p* < 0.05). Uppercase letters compare the means of each species at each lead concentration, while lowercase letters compare the means of each species at the same lead concentration. Vertical lines represent the standard error.

It can be observed that, in relation to transpiration (E), for *I. vera* subsp. *affinis*, there was a decrease in the treatment of 200 mg dm^−3^ Pb (4.01 ± 0.07) in relation to the control (4.94 ± 0.13). In relation to *I. laurina*, there was greater transpiration in the highest dose of Pb (500 mg dm^−3^) (3.58 ± 0.18), with the lowest rate being in the 100 mg dm^−3^ treatment (2.07 ± 0. 10). As for Ingás in the same treatment, it can be observed that *I. vera* subsp. *affinis* has a higher transpiration rate (4.51 ± 0.08) than *I. laurina* (2.97 ± 0.12) in all treatments (Figure 1A).

#### 2.1.2. Photosynthetic Rate (A)

Regarding the photosynthetic rate (A), for *I. vera* subsp. *affinis*, the highest rate is in the treatment with 400 mg dm^−3^ of Pb (10.37 ± 0.11), when compared to the control (7.14 ± 0.33). For *I. laurina*, the highest rate was in the treatment with 200 mg dm^−3^ (8.73 ± 0.13) and the lowest was in the treatment with 100 mg dm^−3^ of Pb (5.09 ± 0.13). In relation to Ingás in the same treatment, *I. vera* subsp. *affinis* has a higher photosynthetic rate (7.77 ± 0.26; 7.87 ± 0.18; 10.37 ± 0.11; 8.17 ± 0.21) compared to *I. laurina* (5.09 ± 0.13; 6.68 ± 0.17; 6.53 ± 0.16) in treatments with 100, 400, and 500 mg dm^−3^ of Pb (Figure 1B).

#### 2.1.3. Water Use Efficiency (WUE)

It can be observed that, regarding water use efficiency (WUE), for *I. vera* subsp. *affinis*, there was a decrease in the treatment with 500 mg dm^−3^ (1.63 ± 0.16) compared to the treatment with 400 mg dm^−3^ (2.23 ± 0.07). For *I. laurina*, the highest efficiency rate was observed with 400 mg dm^−3^ of Pb (3.21 ± 0.10) compared to the other treatments. Regarding the *Inga* species under the same treatment, it can be noted that *I. vera* subsp. *affinis* exhibited lower WUE in the control, and with 100, 400, and 500 mg dm^−3^ of Pb (1.72 ± 0.08; 1.87 ± 0.12; 2.23 ± 0.07; 1.63 ± 0.16) compared to *I. laurina* (2.25 ± 0.01; 2.50 ± 0.10; 3.21 ± 0.10; 2.15 ± 0.07) (Figure 1C).

#### 2.1.4. Internal CO_2_ Concentration (Ci)

Regarding the internal CO_2_ concentration (Ci), it can be observed that in *I. vera* subsp. *affinis*, the treatment with 400 mg dm^−3^ of Pb (247.33 ± 4.89) resulted in a higher Ci compared to the control, 100, and 300 mg dm^−3^ (208.33 ± 10.16; 208.06 ± 7.87; 203.33 ± 16.44). In *I. laurina*, there were no significant differences in Ci between treatments. Comparing the *Inga* species under the same treatment, *I. vera* subsp. *affinis* showed a lower concentration (220.57 ± 4.92) compared to *I. laurina* (250.14 ± 2.16) across all treatments, except for the 400 mg dm^−3^ Pb treatment (247.33 ± 4.89; 247.75 ± 5.50), where no significant differences were observed (Figure 1D).

#### 2.1.5. External CO_2_ Concentration (Ca)

The external CO_2_ concentration (Ca) for *I. vera* subsp. *affinis* increased linearly with rising Pb concentrations in the substrate (305.20 ± 10.60 at the highest concentration). For *I. laurina*, there were no significant differences between treatments. Comparing the Inga species under the same treatment, *I. vera* subsp. *affinis* exhibited a lower concentration up to the 300 mg dm^−3^ treatment (217.45 ± 25.00; 278.73 ± 2.46; 292.23 ± 3.06; 317.48 ± 20.16) compared to *I. laurina* (372.68 ± 0.96; 378.35 ± 0.48; 368.17 ± 0.35; 372.03 ± 0.49) (Figure 1E).

#### 2.1.6. Ratio Between Internal and External CO_2_ Concentrations (Ci/Ca)

There were no significant differences for *I. vera* subsp. *affinis* and *I. laurina* between treatments, nor within the same treatment when comparing the two species (Figure 1F).

#### 2.1.7. Stomatal Conductance (gs)

Regarding stomatal conductance (gs), it can be observed that *I. vera* subsp. *affinis* exhibited the highest value in the treatment with 500 mg dm^−3^ of Pb (221.33 ± 5.83), compared to the treatments with 100 and 200 mg dm^−3^ (154.00 ± 6.30; 155.00 ± 0.55). Similar results were observed for *I. laurina*, where the treatment with 500 mg dm^−3^ showed the highest gs value (133.00 ± 7.41), compared to the treatment with 100 mg dm^−3^ (65.25 ± 2.90). Comparing the Inga species under the same treatment, it can be seen that *I. vera* subsp. *affinis* has higher gs (179.26 ± 5.21) than *I. laurina* (103.90 ± 4.96) in all treatments (Figure 1G).

### 2.2. Determination of Chlorophyll, Carotenoid, and Phenolic Compound Content

#### 2.2.1. Chlorophyll a (Chla)

**Figure 2:**
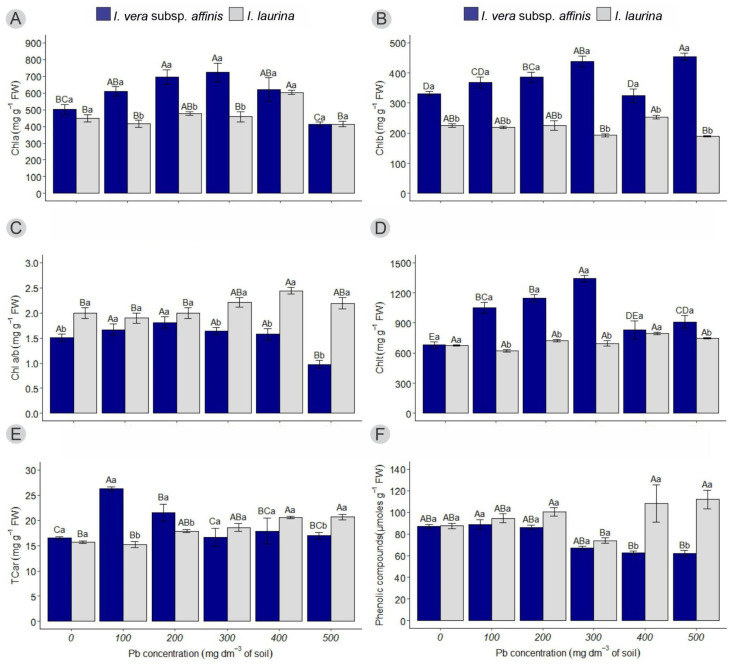
The average of treatments at different lead concentrations for (**A**) chlorophyll a, (**B**) chlorophyll b, (**C**) chlorophyll a/b, (**D**) total chlorophyll, (**E**) total carotenoids, and (**F**) phenolic compounds. Different letters indicate a significant difference by Tukey’s test (*p* < 0.05). Uppercase letters compare the means of each species at each lead concentration, while lowercase letters compare the means of each species at the same lead concentration. Vertical lines represent the standard error.

In *I. vera* subsp. *affinis*, a reduction in chlorophyll a (Chla) content can be observed in the control and 500 mg dm^−3^ Pb treatments (501.16 ± 29.53; 412.69 ± 13.34) compared to the treatments with 200 and 300 mg dm^−3^ (695.35 ± 42.69; 723.02 ± 56.20). In *I. laurina*, the highest Chla content was found in the treatment with 400 mg dm^−3^ (603.93 ± 13.33) compared to the control (448.32 ± 21.31). Regarding the Inga species under the same treatment, higher Chla content was observed in *I. vera* subsp. *affinis* in the 100, 200, and 300 mg dm^−3^ treatments (608.83 ± 28.63; 695.35 ± 42.69; 723.02 ± 56.20) compared to *I. laurina* (415.62 ± 21.08; 477.51 ± 12.18; 457.87 ± 31.23) (Figure 2A).

#### 2.2.2. Chlorophyll b (Chlb)

In *I. vera* subsp. *affinis*, the chlorophyll b (Chlb) content was higher in the treatment with 500 mg dm^−3^ (453.72 ± 12.46) compared to the control (331.80 ± 6.74). For *I. laurina*, the highest Chlb content was observed in the 400 mg dm^−3^ treatment (254.15 ± 6.05), compared to the treatments with 300 and 500 mg dm^−3^ (192.60 ± 5.69; 189.35 ± 2.18). When comparing the Chlb content between the Inga species, it can be observed that in *I. vera* subsp. *affinis*, the content is higher (383.83 ± 10.91) than in *I. laurina* (217.61 ± 5.02) across all treatments (Figure 2B).

#### 2.2.3. Chlorophyll a/b Ratio (Chl a/b)

In *I. vera* subsp. *affinis*, there were no significant differences in the chlorophyll a/b ratio (Chl a/b) from the control up to the treatment with 400 mg dm^−3^ (1.51 ± 0.07). Only at the highest Pb dose was there a decrease (0.98 ± 0.08) compared to the previous treatments. For *I. laurina*, the highest Chl a/b was found in the treatment with 400 mg dm^−3^ (2.44 ± 0.07) compared to the control (2.00 ± 0.11). When comparing the two species, the Chl a/b ratio was lower in *I. vera* subsp. *affinis* (1.51 ± 0.07; 1.64 ± 0.07; 1.57 ± 0.11; 0.98 ± 0.08) than in *I. laurina* (2.00 ± 0.11; 2.21 ± 0.09; 2.44 ± 0.07; 2.19 ± 0.11) in the control, 300, 400, and 500 mg dm^−3^ Pb treatments, with no significant difference in the previous treatments (Figure 2C).

#### 2.2.4. Total Chlorophyll (Chlt)

Regarding total chlorophyll (Chlt), it can be observed that in *I. vera* subsp. *affinis*, the treatment with 300 mg dm^−3^ (1342.69 ± 32.28) resulted in the highest content compared to the control (681.51 ± 30.38). For *I. laurina*, there were no statistical differences with the increase in Pb concentrations (709.29 ± 11.53). Regarding Chlt content between the *Inga* species under the same treatment, the highest content was observed in *I. vera* subsp. *affinis* (1053.84 ± 52.67; 1147.67 ± 34.05; 1342.69 ± 32.28; 911.28 ± 61.16) in the treatments with 100, 200, 300, and 500 mg dm^−3^, compared to *I. laurina* (620.32 ± 12.39; 722.57 ± 12.01; 694.68 ± 27.13; 746.79 ± 5.02) (Figure 2D).

#### 2.2.5. Total Carotenoids (TCar)

In *I. vera* subsp. *affinis*, the total carotenoid content (TCar) was higher in the treatment with 100 mg dm^−3^ of Pb (26.31 ± 0.37) compared to the control (16.48 ± 0.32). In *I. laurina*, the highest TCar was observed at the higher Pb concentrations (400 and 500 mg dm^−3^) (20.53 ± 0.25; 20.66 ± 0.54) compared to the control (15.70 ± 0.24). Regarding the TCar between the Inga species under the same treatment, higher TCar was observed in *I. vera* subsp. *affinis* in the treatments with 100 and 200 mg dm^−3^ (26.31 ± 0.37; 21.54 ± 1.71) compared to *I. laurina* (15.21 ± 0.55; 17.87 ± 0.33) (Figure 2E).

#### 2.2.6. Phenolic Compounds

It can be observed that *I. vera* subsp. *affinis* accumulated more phenolic compounds in the treatment with 100 mg dm^−3^ (88.85 ± 4.45) compared to the treatments with 400 and 500 mg dm^−3^ of Pb (62.36 ± 1.68; 62.18 ± 2.43). In *I. laurina*, a lower accumulation of phenolic compounds was observed in the treatment with 300 mg dm^−3^ (73.87 ± 2.42) compared to the treatments with 200, 400, and 500 mg dm^−3^ (11.37 ± 3.80; 108.29 ± 17.32; 111.90 ± 8.54). Regarding the phenolic compound content between the Inga species under the same treatment, a lower amount of phenolic compounds was observed in *I. vera* subsp. *affinis* (62.36 ± 1.68; 62.18 ± 2.43) compared to *I. laurina* (108.29 ± 17.32; 111.90 ± 8.54) in the treatments with 400 and 500 mg dm^−3^, with no significant differences in the previous treatments (Figure 2F).

### 2.3. Anatomical Characterization

**Figure 3:**
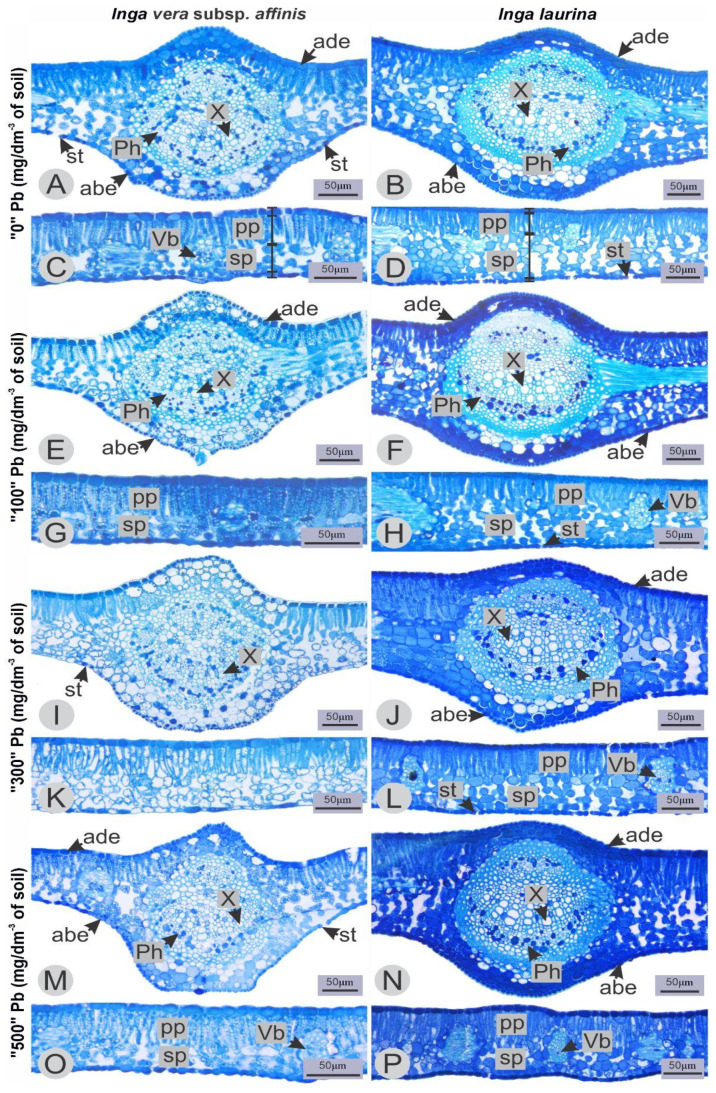
Leaf anatomy of *Inga vera* subsp. *affinis* and *Inga laurina* under different lead treatments in the soil. (**A**,**C**,**E**,**G**,**I**,**K**,**M**,**O**): *Inga vera* subsp. *affinis.* (**B**,**D**,**F**,**H**,**J**,**L**,**N**,**P**): *Inga laurina*. (**A**–**D**): control. (**E**–**H**): Treatment with 100 mg Pb/dm^3^ of soil. (**I**–**L**): treatment with 300 mg Pb/dm^3^ of soil. (**M**–**P**): treatment with 500 mg Pb/dm^3^ of soil. abe: abaxial surface. ade: adaxial surface. Ph: Phloem. pp: palisade parenchyma. sp: spongy parenchyma. st: stomata. Vb: vascular bundle. X: Xylem.

Regarding the characterization of the leaf mesophyll, both species are dorsiventral. The adaxial and abaxial epidermis of *I. vera* subsp. *affinis* (Figure 3A,E,I,M) and *I. laurina* (Figure 3B,F,J,N) are both uniseriate. The palisade parenchyma is also unicellular and the lacune parenchyma contains two to three layers of cells in *I. vera* subsp. *affinis* (Figure 3C,G,K,O) and four to five layers in *I. laurina* (Figure 3D,H,L,P).

From the statistical analyzes it was observed that, for each of the two species, the Pb concentrations used in this study did not change any of the five tissues measured compared to the control (Table 1).

**Figure 4:**
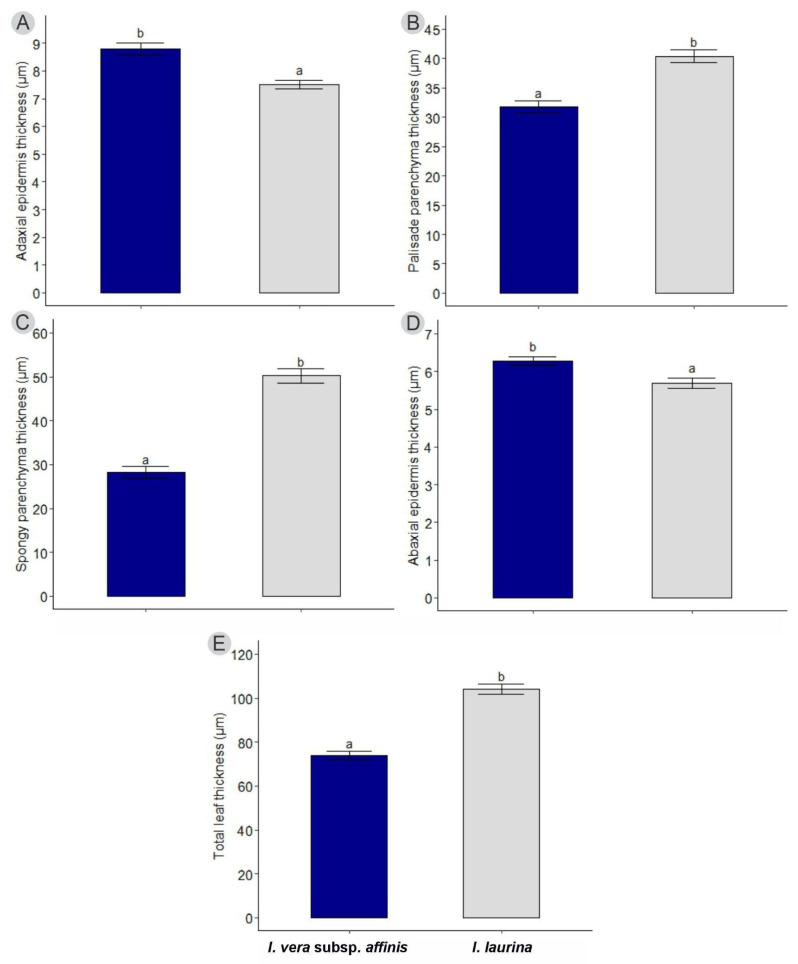
The average of the morphometric measurements of the leaf tissues of *Inga vera* subsp. *affinis* (blue bar) and *Inga laurina* (gray bar). (**A**) Thickness of the adaxial epidermis, (**B**) thickness of the palisade parenchyma, (**C**) thickness of the spongy parenchyma, (**D**) thickness of the abaxial epidermis, and (**E**) total leaf thickness. Vertical lines represent the standard error. Different letters indicate a significant difference between the mean of the species analyzed at 5% significance.

However, when comparing the two species, significant differences were observed in all tissues analyzed. The species *I. vera* subsp. *affinis* presented larger adaxial (ade) and abaxial (abe) epidermises with means of 8.79 ± 0.20 and 6.27 ± 0.11, respectively (Figure 4A,D). While the species *I. laurina* presented greater palisade (pp), lacunous (sp) parenchyma and total leaf thickness (tlt) with averages of 40.38 ± 1.07, 50.22 ± 1.57, and 104.17 ± 2.31, respectively (Figure 4B,C,E).

## 3. Discussion

The analyzed species presented variations in gas exchange data and photosynthetic pigments in relation to Pb concentrations in the substrate. However, these parameters were not substantially affected by the sharp increase in this metal concentration. Anatomically, no significant changes in leaf tissues were observed in response to lead, so that these results—combined with the physiological data—suggest a possible tolerance from the two evaluated Ingá species to the conditions of exposure to lead.

Regarding gas exchange, even at the highest Pb concentrations (400 and 500 mg dm^−3^), both Ingá species (*I. vera* subsp. *affinis* and *I. laurina*) were able to maintain their photosynthetic apparatus active even in soils with high Pb concentrations. Elements such as Cd, Ni, and Pb promote stomatal closure, resulting in photosynthesis inhibition [22], while lead (Pb) affects photosynthesis by inhibiting Rubisco activity, promoting enzymatic inhibition, water imbalance with reduced water use efficiency, and changes in membrane permeability, in addition to the formation of ROS [23]. However, in the present study, the Pb presence was not able to affect photosynthetic parameters (even at the highest Pb concentrations in the substrate) of photosynthetic rate (A), transpiration (E), water use efficiency (WUE), internal carbon concentration (Ci), and external carbon concentration (Ca), for both species. For *I. vera* subsp. *affinis* and *I. laurina*, similar behavior in photosynthetic parameters can be explained by the absence of metal allocation in the leaves, as presented in previous works [20].

Heavy metals such as Pb can reduce cell wall plasticity by causing a reduction in the guard cell turgor: changes in cell water potential and problems in regulating the opening and closing of stomata. Guard cells close their stomata due to turgor pressure loss [24]. Some of the direct toxic effects caused by high metal concentration include inhibition of cytoplasmic enzymes and damage to cellular structures due to oxidative stress and pigment reduction [25]. When gas exchange was evaluated, we observed that even at the highest Pb concentration (500 mg dm^−3^), *I. vera* subsp. *affinis* was able to maintain high parameters in the opening and closing of stomata (gs). In general terms, the species *I. vera* subsp. *affinis* managed to maintain its photosynthetic metabolism with the metal presence, indicating that this species is capable of growing even in soils with excess Pb. In contrast, when it was studied, the consequences of *Morus alba* L. (mulberry) exposure to Pb revealed that high concentrations of the metal caused a reduction in the transpiration rate [23]. This same result was obtained by Zulfiqar [24], where the presence of Pb (500 and 2000 mg kg^−1^ of Pb) reduced transpiration rates in *Plantago major.* The distinct responses between *I. vera* subsp. *affinis* and *I. laurina*, although they are from the same genus, can be attributed to their occurrence in different niches; therefore, the species respond differently to stresses.

When evaluating the chlorophyll levels, we observed that even with the highest Pb concentration in the substrate, the species *I. vera* subsp. *affinis* was able to maintain a high content of chlorophyll a and chlorophyll b. This fact explains the positive effect on the photosynthetic rate (Figure 1), showing that plants are tolerant to excess Pb and are able to complete their cycle even under adverse conditions. For the *I. laurina* species, in the highest Pb concentrations, high levels of carotenoids and phenolic compounds were observed. In general, the increase in carotenoid levels in plants is related to increased tolerance to oxidative stress [26]. When it was studied, the phenolic metabolism of *Kandelia obovata* under Cd and Zn stress experienced an increase in phenolic compounds [27].

The first consequence of heavy metal toxicity in plant cells is ROS overproduction [28]. Understanding plant tolerance to oxidative stress is essential for evaluating the development of different species, since stress can interfere with their metabolism, influencing their development and preventing increased production. According to results from similar works, in soils with high Pb concentrations, *Inga* species can be used to assist in phytoremediation, since the results obtained showed higher transpiration (E) and stomatal conductance (gs) rates, indicating the absence of toxicity in the leaves, a consequence of the Ingá phytostabilizing characteristic [20], since approximately 95% of the absorbed Pb is accumulated in the roots and a small amount is translocated to the shoots [29].

Additionally, in the *I. vera* subsp. *affinis* and *I. laurina* leaf tissues, no changes in thickness were observed in response to the increasing Pb concentrations evaluated in this study. However, in another species of the genus, *I. subnuda* subsp. *luschnathiana* (Benth.) TDPenn., an increase in the adaxial epidermis and a decrease in the abaxial epidermis were reported at concentrations of 0.36 mmol L^−1^ of Cu [30], indicating that for this genus, anatomical responses may vary according to the metal and also the allocation organ [20]. In the Pb case, the 100 µM Pb^2+^ concentration has increased the thickness of tissues such as palisade and lacunar parenchyma and adaxial epidermis in *Cichorium intybus* L. var. *intybus* [13], as well as increased the epidermis of *Schinus molle* L. [15]; however, the effects of each metal may vary according to the plant species.

The two species analyzed in this study are present from Central to South America [31] and both cover various types of vegetation, such as coastal zones and dry forests [32]. Among the Brazilian biomes, *I. vera* subsp. *affinis* can be found in phytophysiognomies such as Cerrado (lato sensu), ciliary or gallery forest, igapó forest, terra firme forest, floodplain forest, semideciduous seasonal forest and *I. laurina* in semideciduous seasonal forest, ombrophilous forest (rainforest), and restinga [32]. The differences in tissue structure between the two species may possibly be related to the ecological niche and their evolutionary history, so future studies that address this issue would be interesting.

## 4. Materials and Methods

### 4.1. Soil Characteristics and Collection Site

The soil used was oxisol, originating from the Cerrado biome, collected from the area belonging to the Teaching, Research, and Extension Farm of UNESP at Ilha Solteira, located in the municipality of Selvíria-MS, Brazil (20°20′35″ S and 51°24′04″ W, at an altitude of 358 m). The chemical conditions of the soil [33] before the experiment was implemented showed high acidity and low base saturation (pH in CaCl_2_ of 4.4 and V% of 16%), base sum (SB) of 16.4 mmolc dm^−3^, Ca^2+^ of 7 mmolc dm^−3^, K^+^, and Mg^2+^ of 2.4 and 7 mmolc dm^−3^, respectively, potential acidity (H + Al) of 88 mmolc dm^−3^, with 28 g dm^−3^ of organic matter (OM), and cation exchange capacity (CEC) of 104.4 mmolc dm^−3^. For micronutrients, the concentrations were 32.8, 32, 2.5, and 1.5 mg dm^−3^ for Mn, Fe, Cu, and Zn, and the B concentration was 0.39 mg dm^−3^. The granulometric analysis shows that the soil is composed of 426 g kg^−1^ of clay, 502 g kg^−1^ of total sand, and 72 g kg^−1^ of silt. Subsequently, a volume of 2 dm^3^ of the soil–sand mixture (1:1 substrate) was placed in plastic bags with a capacity of 2 L for the artificial contamination procedure to be performed.

### 4.2. Soil Contamination and Experiment Setup

The experiment was conducted in a greenhouse at the Laboratory of Plant Metabolism Physiology (LFMV) at UNESP Ilha Solteira Campus, São Paulo, Brazil, from 8 April 2019 to 4 April 2020, using a randomized block design. (The methodology of a completely randomized block design (CRBD) involves dividing the experimental units into groups called blocks, where each block represents a source of controlled variation, such as different environmental conditions or soil characteristics. Each block consists of experimental units that are similar to each other but may vary between blocks. Within each block, the treatments are randomly assigned to the experimental units, ensuring that the treatment distribution is unbiased and free from any systematic error. The experiment is carried out by applying the treatments according to the defined randomization, and data on the dependent variables are collected as per the established protocol.) For the following experiment, a substrate of soil (oxisol) and sand was used, in a 1:1 ratio, to increase soil porosity. For the artificial contamination of the substrate (2 dm^3^), lead acetate (Pb (CH_3_COO)_2_), which has high solubility [34], was diluted in 300 mL of water, forming the doses of 100, 200, 300, 400, and 500 mg Pb/dm^−3^.

According to CETESB [35], the reference value for Pb in the soil is 17 mg kg^−3^ and the preventive value is 72 mg kg^−3^. For agricultural and residential areas, the values are 150 and 240 mg dm^−3^, respectively, which are considered high and require intervention. A volume of 2 dm^3^ of the substrate was placed in transparent plastic bags, which were labeled with the treatment number and replication, corresponding to the experimental units. The Pb contaminant solutions were added to each experimental unit’s plastic bag in a quantity of 20 mL. For the control, 20 mL of distilled water was added. Then, the soil was manually homogenized and incubated for 30 days in the greenhouse, without watering or exposure to sunlight, to allow the metal to stabilize in the soil. After the stabilization period, a composite soil sample was collected from each treatment and a physical–chemical analysis was carried out, using the USEPA (U.S Environmental Protection Agency) method, with reading on the ICP–AES (Inductively Coupled Plasma–Atomic Emission Spectrometry) (EPA 6010c) [36], to verify the available Pb levels [20].

Subsequently, 6-month-old seedlings of *I. vera* subsp. *affinis* and *I. laurina*, commercially acquired from the Flora Londrina nursery in Londrina/PR, were transplanted into the stabilized soil, receiving automatic irrigation three times per day, with a duration of 5 min each. The experiment was conducted using a completely randomized design (CRD) by species, that is, one CRD for *I. vera* and another for *I. laurina*, run concurrently. Each CRD contained 6 treatments (control and Pb doses), with 5 repetitions, totaling 30 experimental units per species.

### 4.3. Photosynthetic Parameters

The leaf gas exchange measurement parameters were performed at 60 days of cultivation, in the vegetative stage (before the collection of the experiment). The measurement was conducted on fully expanded and apparently healthy leaves in the central region of the leaf blade during the vegetative phenological stage, between 09:00 and 12:00 h in the morning, at an average temperature of 35 °C. To determine the photosynthetic response of these plants, measurements of photosynthesis parameters were performed to identify potential changes in response to metal exposure, using a portable gas exchange analyzer, CIRAS-3 (Portable Photosynthesis System-PP Systems).

The following parameters were analyzed: transpiration (E, mol H_2_O m^−2^ s^−1^); photosynthetic rate (A, µmol CO_2_ m^−2^ s^−1^); water use efficiency (WUE = A/E); internal CO_2_ concentration (Ci, µmol mol^−1^); external CO_2_ concentration (Ca, µmol mol^−1^); the ratio of internal to external CO_2_ concentrations (Ci/Ca); and stomatal conductance (gs, mol H_2_O m^−2^ s^−1^).

### 4.4. Collection

After 8 months of cultivation, specifically 240 days, the plants were removed from the substrate bags, washed with distilled water, and dried with paper towels. The leaves were separated, with part allocated for physiological analyses and the other part for morphological analyses.

### 4.5. Determination of Chlorophyll and Carotenoid Content

Thin strips (1 mm) of leaf were cut to obtain 50 mg, and the material was then placed in a test tube with 7 mL of DMSO (or 25 mg + 3.5 mL DMSO). The mixture was subsequently placed in a water bath at 65 °C for 30 min and then cooled in the dark for reading in a spectrophotometer to determine the chlorophyll content (645 and 663 nm) and carotenoid content (480 nm) [37].

### 4.6. Determination of Total Phenolic Compounds Content

The leaves were macerated, followed by the addition of 200 μL of the sample to 1 mL of 0.25 Folin–Ciocalteu reagent. After 4 min, 800 µL of a saturated sodium carbonate solution (approximately 75 g L^−1^) were added. After 2 h at room temperature, the absorbance of the solution was measured at 760 nm. Gallic acid (0–500 mg L^−1^) was used to calibrate the standard curve. The total phenolic content was expressed in milligrams of gallic acid equivalent (mg GAE g^−1^) per dry weight of the plant material [38].

### 4.7. Anatomical Characterization

For the anatomical analysis, leaves were collected from 5 different individuals (replicates) only from the treatments 0, 100, 300, and 500 mg dm^−3^ of Pb. Such treatments were selected to encompass control, lower dose, and higher doses.

The leaves were fixed in FAA 70% [39] to avoid loss of material due to natural degradation, and after 48 h the material was stored in 70% alcohol. In the completely expanded leaf, the median region of the leaf blade was analyzed. The samples were dehydrated in an ethyl series, embedded in hydroxyethyl methacrylate (Leica Historesin, Leica Microsystems, Wetzlar, Germany), and the blocks obtained were sectioned with a thickness of 5–10 µm. The material was stained with 0.05% Toluidine Blue in phosphate buffer and citric acid pH between 4.5 and 6.0 [40], and the slides were mounted in synthetic resin “Entellan”.

Digital photomicrographs were obtained with the AxioCam ERC5S Zeiss Primo Star microscope (Carl Zeiss Microscopy GmbH, Göttingen, Germany), with the micrometric scales photographed and enlarged under the same optical conditions.

From the samples obtained from the 5 individuals per treatment, 5 histological slides were prepared for each. Thus, 2 photomicrographs were obtained from each of them. These photomicrographs were submitted to the image processing program Image J 1.54g [41], which allows measuring tissues in micrometers (µm). To assess whether lead altered the structure, the following tissues were measured on the leaf blade, in a region free of vascular bundles: adaxial epidermis (ade), palisade parenchyma (pp), spongy parenchyma (sp), abaxial epidermis (abe), and total leaf thickness (tlt).

### 4.8. Statistical Analysis

The data analyses were performed using R 4.3.0 software (Package ExpDes.pt) and ggplot2. The normality hypothesis was tested using the Shapiro–Wilk test, and the homogeneity of variances was evaluated using Bartlett’s test. When ANOVA indicated a significant difference between treatments, the means of the variables were compared using the Tukey test at a 5% probability level.

## 5. Conclusions

*I. vera* subsp. *affinis* and *I. laurina* maintained stable photosynthetic parameters, even under high concentrations of lead (Pb). Regarding photosynthetic pigments, *I. vera* subsp. *affinis* presented high levels of chlorophyll a and chlorophyll b, even at the highest Pb concentration. Species *I. laurina*, in turn, demonstrated greater accumulation of carotenoids and phenolic compounds at higher Pb concentrations in the substrate. In leaf tissues, there were also no structural changes in cell thickness or shape. The observed differences in the tissues refer to the comparison between the species, but not in relation to Pb. These results suggest that both species have heavy metal stress adaptation mechanisms, allowing the maintenance of photosynthetic activity, ensuring that the species complete their life cycles under adverse conditions.

Greenhouse experiments for short periods are important as pilot studies to identify species with potential tolerance to metal stress. In field trials, as there is no temperature and irrigation control, there may be changes in the results, making it necessary to cultivate and evaluate the species in the long term to validate how the plant responds to stress over time.

## Figures and Tables

**Table 1:** Averages with associated standard error of the leaf tissues: adaxial epidermis, palisade parenchyma, spongy parenchyma, abaxial epidermis, and total leaf thickness (μm) of *Inga vera* subsp. *affinis* and *Inga laurina* cultivated in lead-contaminated soil (mg dm^−3^ of soil). Uppercase letters compare the means of each species at each lead concentration, while lowercase letters compare the means of each species at the same lead concentration. Pr > Fc: significance associated with the treatment. * *p* < 0.05.

Thickness (µm)					Source of Variation
Total Leaf	Abaxial Epidermis	Spongy Parenchyma	Palisade Parenchyma	Adaxial Epidermis	
0.528 ns	0.870 ns	0.717 ns	0.909 ns	0.871 ns	Pr > Fc Pb concentration
0.000 *	0.002 *	0.000 *	0.000 *	0.000 *	Pr > Fc Species
0.277 ns	0.221 ns	0.075 ns	0.929 ns	0.614 ns	Pr > Fc Pb concentration * Species
39.23		36.10		8.15	Mean
15.78		13.70		10.26	CV (%)
Means					Pb concentration * Species
73.49 ± 5.71 Aa	5.94 ± 0.17 Aa	26.67 ± 2.43 Aa	32.42 ± 2.68 Aa	8.82 ± 0.64 Aa	0 * *I. vera* subsp. *affinis*
74.63 ± 2.65 Aa	6.38 ± 0.15 Aa	27.91 ± 1.34 Aa	31.70 ± 1.29 Aa	8.72 ± 0.33 Aa	100 * *I. vera* subsp. *affinis*
72.04 ± 4.52 Aa	6.30 ± 0.27 Aa	27.11 ± 2.70 Aa	30.51 ± 2.53 Aa	8.55 ± 0.38 Aa	300 * *I. vera* subsp. *affinis*
75.67 ± 2.07 Aa	6.47 ± 0.24 Aa	31.29 ± 3.62 Aa	32.65 ± 1.58 Aa	9.09 ± 0.32 Aa	500 * *I. vera* subsp. *affinis*
111.55 ± 4.09 Aa	5.94 ± 0.41 Aa	55.68 ± 3.81 Aa	41.40 ± 1.15 Aa	7.60 ± 0.20 Aa	0 * *I. laurina*
103.80 ± 6.02 Aa	5.83 ± 0.19 Aa	49.21 ± 3.32 Aa	40.63 ± 3.08 Aa	7.82 ± 0.21 Aa	100 * *I. laurina*
104.14 ± 1.63 Aa	5.54 ± 0.27 Aa	50.70 ± 1.79 Aa	40.06 ± 2.58 Aa	7.40 ± 0.14 Aa	300 * *I. laurina*
97.21 ± 4.47 Aa	5.47 ± 0.14 Aa	45.29 ± 2.15 Aa	39.44 ± 1.96 Aa	7.22 ± 0.50 Aa	500 * *I. laurina*

## Data Availability

Data will be made available on request.
